# Neuroanatomical Pattern of Mitochondrial Complex I Pathology Varies between Schizophrenia, Bipolar Disorder and Major Depression

**DOI:** 10.1371/journal.pone.0003676

**Published:** 2008-11-07

**Authors:** Dorit Ben-Shachar, Rachel Karry

**Affiliations:** Laboratory of Psychobiology, Department of Psychiatry Rambam Medical Center and B. Rappaport Faculty of Medicine Technion, Haifa Israel; Chiba University Center for Forensic Mental Health, Japan

## Abstract

**Background:**

Mitochondrial dysfunction was reported in schizophrenia, bipolar disorderand major depression. The present study investigated whether mitochondrial complex I abnormalities show disease-specific characteristics.

**Methodology/Principal Findings:**

mRNA and protein levels of complex I subunits NDUFV1, NDUFV2 and NADUFS1, were assessed in striatal and lateral cerebellar hemisphere postmortem specimens and analyzed together with our previous data from prefrontal and parieto-occipital cortices specimens of patients with schizophrenia, bipolar disorder, major depression and healthy subjects. A disease-specific anatomical pattern in complex I subunits alterations was found. Schizophrenia-specific reductions were observed in the prefrontal cortex and in the striatum. The depressed group showed consistent reductions in all three subunits in the cerebellum. The bipolar group, however, showed increased expression in the parieto-occipital cortex, similar to those observed in schizophrenia, and reductions in the cerebellum, yet less consistent than the depressed group.

**Conclusions/Significance:**

These results suggest that the neuroanatomical pattern of complex I pathology parallels the diversity and similarities in clinical symptoms of these mental disorders.

## Introduction

The past decade has witnessed an abundance of studies focusing on mitochondrial abnormalities in several mental disorders including schizophrenia, bipolar disorder and major depression. The role mitochondria play in mental disorders has been investigated using a wide array of experimental techniques ranging from imaging studies through ultrastructural methods to genetic and molecular means.

Imaging studies using phosphorous magnetic resonance spectroscopy (^31^P-MRS) and ^1^H- MRS demonstrated reduced mitochondrial originated high energy phosphates, such as ATP and phosphocreatine (PCr) as well as other cellular factors whose metabolism is strongly suggested to be linked to mitochondrial ATP production, in schizophrenia relevant brain structures of schizophrenic patients [1]–[8]. In bipolar disorder similar mitochondrial abnormalities have been reported [9]–[11], while in major depression, the current literature on MRS studies is sparse and inconsistent [12]–[14].

Genetic studies also implicate mitochondria abnormalities in schizophrenia and in affective disorders. For example, two single nucleotide polymorphisms (SNPs) in a nuclear encoded subunit of complex I, NDUFV2, were found to be associated with schizophrenia and with bipolar disorder [15], [16]. Additional genetic variations in mitochondrial DNA encoded ND3 and ND4 subunits of complex I were associated with bipolar disorder and schizophrenia, respectively [17], [18]. These studies suggest the genetic variation in complex I as a risk factor in both disorders.

Finally, accumulating molecular, transcriptomic, proteomic and metabolomic approaches as well as biochemical data points to abnormalities in mitochondria in both periphery and brain in schizophrenia [19]–[28]. Focusing on the mitochondrial oxidative phosphorylation system (OXPHOS) in schizophrenia, revealed alterations in the enzymatic activities of complexes IV, II and I–III and in mRNA and protein levels of complex I subunits, NDUFV1 and NDUFV2, in post-mortem brain specimens [26], [29]–[32]. Similarly, alterations both in complex I activity and its subunit expression were observed in peripheral blood cells of schizophrenic patients [22], [27], [33], [34]. In bipolar disorder a reduction in the expression level of mitochondrial genes, including those of the OXPHOS was observed in hippocampal and prefrontal postmortem specimens [35]–[37], while an increase in complex I subunits NDUFV1 and NDUFV2 was observed in the parieto-occipital cortex [26]. In major depression, although most studies did not show cortical modifications in mitochondrial related genes, some reports suggest alterations in the expression of nuclear as well as in mitochondrial DNA encoded genes in the prefrontal cortex [26], [37]. In addition, it was demonstrated that muscle mitochondria in depressed patients produced less ATP and that the activity of the OXPHOS complexes I+III and II+III was impaired [38].

The studies described hitherto suggest a dysregulation of mitochondrial function in schizophrenia and mood disorders, consequently raising the question as to whether mitochondrial impairment displays disease-specific characteristics or is rather a general non-distinguishing pathology of these disorders. Complex I, the focus of the present study, plays a major role in controlling oxidative phosphorylation, and therefore mitochondrial function [39]. The aim of the present study was to determine whether complex I abnormalities show disease-specific characteristics. mRNA and protein levels of three subunits of complex I, NDUFV1, NDUFV2 and NDUFS1, all forming one functional subunit, were assessed in postmortem brain specimens of striatum and cerebellum of patients with schizophrenia, bipolar disorder or major depression and normal subjects, and analyzed together with our previous data from prefrontal and parieto-occipital cortices of the same cohorts. Tables 1, 2, 3 summarize the main clinical, functional, biochemical and pathogenic characteristics of the different mental disorders, brain areas and complex I subunits investigated in the present study. The abnormality in mitochondrial complex I subunits' parameters demonstrated disease-specific regional distribution discriminating between schizophrenia, bipolar disorders and major depression.

**Table 1:** Clinical characteristics of the three different mental disorders investigated in the present study.

Disease	Population prevalence	Typical age of onset	Gender differences	Symptoms according to the Diagnostic and statistical Manual of Mental Disorders –DSM-IV
Schizophrenia	1.1% ^a^	M – 18 yr F – 25 yr	F = M	The essential features of Schizophrenia are a mixture of characteristic signs and symptoms (both positive and negative) that have been presented for a significant portion of time during a 1 month period with some signs persisting for at least 6 months. These signs include cognitive, emotional and behavioral anomalies.
				Positive symptoms: delusions, hallucinations, disorganized speech (eg. frequent derailment or incoherence), grossly disorganized or catatonic behavior, Negative symptoms, i.e. affective flattening, alogia or avolition.
				Two or more of these symptoms each present
Bipolar disorder (BP)	2.6% ^a^	20–35 yr	F = M	The essential feature of BP is the occurrence of one or more Manic episodes. Often individuals have on or more MD episodes whose symptoms are summarized for MD.
				Manic episode: inflated self-esteem or grandiosity, decreased need for sleep, flight of ideas, distractibility, increase in goal-directed activity or psychomotor agitation, excessive involvement in pleasure activitiesthat have high potential of painful consequences.
				Three or more of these symptoms lasting for at least 1 week.
Major depression (MD)	5.3% ^a^	30–40 yr with wide variations	F>M	The essential feature of MD is a period of at least 2-weeks during which there is either depressed mood or lost of interest or pleasure in nearly all activities.
				Five or more of the following symptoms presented during 2-weeks and represent change from previous functioning: Depressed mood, markedly diminished interest or pleasure, significant weight loss or weight gain, insomnia or hypersomnia, psychomotor agitation or retardation, fatigue or loss of energy, felling worthless or excessive inappropriate guilt, diminished ability to think or concentrate, recurrent thoughts of death.

**Table 2:** Functional characteristics of the four different brain areas investigated in the present study.

Brain area sections	Location	Function	Reference:
Striatum including the nucleus accumbens		Caudate: motor control, learning and memory (especially feedback processing), language comprehension	[91]–[98]
		Putamen: reinforcement learning	
		Nucleus accumbens: reward, addiction and emotions such as pleasure and laughter, fear, and the placebo effect.	
Cerebellum		Balance, coordination, muscle tension, posture, balance of limbs, fine motion control and eye movement. Recent findings suggest a role in mood and cognition (70)	[99]–[102]
BA 46/9	Dorsolateral prefrontal cortex	Motor planning, organization, and regulation. It plays an important role in the integration of sensory and mnemonic information and the regulation of intellectual function and action and working memory.	[103]–[106]
BA 19	Extrastriate cortex in the occipital cortex	A visual association area, with feature-extracting, shape recognition, attentional, and multimodal integrating functions	[107]–[109]

**Table 3:** Biochemical and pathogenic characteristics of complex I subunits investigated in the present study.

Complex I subunit	Biochemical function	Pathology associated with mutations or polymorhism
NDUFV1	Flavoprotein, contains the NADH-binding site, and binds NADH released electron together with NADUV2.	Leigh-Like syndrome with early onset Ophthalmoplegia (611A→G (Y204C))/ 616T→G (C206G) [112]
	Catalytic sites:	Mitochondrial Complex I Deficiency - 611A→G (Y204C); 616T→G (C206G); 640G→A (E214K);1294G→C (A432P) ; Deletion nt 989–990 [113]
	flavin mono-nucleotide (FMN)	Leukodystrophy and myoclonic epilepsy 175C→T (R59X); 1268C→T (T423M) [114]
	4Fe-4S cluster (N3) [110], [111]	
NDUFV2	Flavoprotein that together with NDUFV1 binds the electron and passes it probably to NDUFS1 Catalytic sites:	Schizophrenia – polymorphisms – rs56506640 (−3542A>G); rs 51156044 (−602G→A) [115]
	2Fe-2S cluster (N1a)[110], [111]	Bipolar disorder – polymorphisms- rs56506640 (−3542A>G); rs 51156044 (−602G→A) [115], [116]
		Early onset hypertrophic cardiomyopathy and encephalopathy- 4-bp deletion- IVS2 +5-+8 (GTTA) [117]
		Parkinson's disease Parkinson's disease – polymorphism (182C→T) [118]
NDUFS1	Iron sulfur protein. The largest transmembrane subunit of complex I.	Mitochondrial Complex I Deficiency –721C→ T (R241W); 755 A→G (D252G); 2119 A→G (M707V);664–666 3 bp deletion 222 [113]
	Catalytic sites:	
	2Fe-2S cluster (N1b)	
	4Fe-4S cluster (N4)	
	and probably 4Fe-4S cluster (N5) [110], [111]	

## Materials and Methods

### Post-mortem tissues

Frozen samples from the striatum including the nucleus accumbens, and the lateral cerebellar hemisphere, were provided by the Stanley Foundation Neuropathology Consortium (Bethesda, MD). Samples were obtained from individuals diagnosed (DSM-IV criteria) with schizophrenia, bipolar disorder or major depression and normal controls 15 subjects in each group. Medication undertaken by each patient is summarized in Table 4. The four groups are matched by age, sex, race, postmortem interval (PMI), pH, laterality, and mRNA quality. Demographics are presented in Table 5. A more detailed description of the Stanley Brain Collection, and protection of human rights is reported in [40]. All 60 samples were analyzed in parallel, blind to patients' diagnosis.

**Table 4:** Summary of medication undertaken by each patient in each diagnostic group.

Psychiatric diagnosis			
Schizophrenia		Bipolar	Major depression
Subject #			
1	Thiothixene, desipramine	Thiothixene, carbamazepine, lithium, trazadone	Imipramine, amitriptyline, nortiptyline, clonazepam
2	None; untreated for over 20 yrs.	Valproate, sertraline, chlorprothixene, carbamazepine	Lithium
3	None; untreated for several months	Lithium, bupropion, clonazepam, lorazepam	Fluoxetine, imipramine, lorazepam
4	None; had ECT but probably never treated otherwise	Lithium, carbamazepine	Phenytion for a single seizure; no other meds for 5 yrs
5	Thioridazine, amitriptyline	Lithium, clozapine	No medication for 6 yrs
6	Clozapine	Never treated	Diphenhydramine, cloxazepam
7	Clozapine	Haloperidol, diphenhydramine	Fluoxetine, lithium
8	Haloperidol, iphenhydramine	Risperidone, valproate, venlafaxine	Nefazadone, hydroxyzine
9	Risperidone, paroxetine	Untreated for over 20 years	Never treated
10	Haloperidol, carbamazepine, fluoxetine, clonazepam, benzotropine	Halperidol, trazadone, trihexphenidyl	Temazepam but off medications for more than 2 months
11	Clozapine, chlorpromazine, lithium	Valproate, bupriopion	Sertraline
12	Haloperidol, lithium, diphenhydramine, chloral hydrate	None, untreated for several months	Venlafaxine, buspirone, alprazolam
13	Clozapine, chlorpromazine, maprotiline, benzotropine, diphenhydramine	Fluoxetine, valproate	None
14	Haloperidol, clozapine, clonazepam	Valproate, clozapine, flurazepam, benzotropine	Trimipramine
15	Risperidone,thioridazine	Valproate, clomipramine	Fluoxetine, nefazadone

**Table 5:** Demographic data for post mortem brains.

Variable	Control (n = 15)	Schizophrenia (n = 15)	Bipolar disorder (n = 15)	Major depression (n = 15)
Age (years, means±S.D.)	48.1±10.7	44.53±13.11	42.3±11.7	46.4±9.3
Gender (male, female)	9M, 6F	9M, 6F	9M, 6F	9M, 6F
Postmortem interval (h, means±S.D.)	23.7±9.94	33.94±14.62	32.5±16.1	27.5±10.7
Cause of death				
*Cardiac*	13	6	4	7
*Accident*	2	2	1	
*Suicide*		4	9	7
*Other*		3	1	1
Age of onset (years, means±S.D.)	N/A	23.20±7.95	33.93±13.29	21.47±8.35
pH (means±S.D.)	6.3±0.2	6.2±0.26	6.2±0.2	6.2±0.2
Brain hemisphere used (right∶left)	8∶7	9∶6	7∶8	9∶6
Lifetime antipsychotic dose^a^ (mg, means±S.D.)	0	52267±62061	20827±24016	0
History of psychosis		15	11 with	
			4 without	
Current alcohol/drug abuse or dependence	0	3	4	3
Past alcohol/drug abuse or dependence	2	3	3	1

a Lifetime antipsychotic dose in fluphenazine milligram equivalents. N/A – not applicable.

### RT-PCR

RNA was extracted from tissue using RNA STAT-60 kit (TEL-TEST, INC, Frienwood, TX,) and treated by DNase as described previously [27], [41]. RNA integrity depicted in the form of three bands corresponding to 28S, 18S and 5S-RNA was assessed by electrophoresis, and its amount and purity was determined spectophotometricaly. The expression of *NDUFV1*, *NDUFV2* and *NDUFS1* encoding for 51-kDa, 24-kDa and 75-kDa subunits of complex I, respectively, was studied by reverse transcriptase-polymerase chain reaction (RT-PCR) analysis. Amplification of RT-cDNA was first performed on a control specimen at different concentrations to define a linear range for all genes. Number of cycles, cDNA amount and primers concentration were established according to a stringent calibration process determining the log-linear phase of amplification for each gene. After establishing the optimal reaction conditions, amplification was performed at least twice for each individual. Sequences of PCR primers for *NDUFV1*, *NDUFV2*, *NDUFS1*, 18S-RNA and β-actin are summarized in Table 6. β-actin and 18S-RNA were used for assessment of RNA quality and yield. β-actin and 18S-RNA were used for assessment of RNA quality and yield. The β-actin was used for normalizing variations in RNA aliquots, as its levels were not affected by disease. A single batch of human platelets RNA, on which PCR was performed at three different concentrations, was assayed in parallel with each set of samples as a positive control, to control for the log-linear phase of amplification for each reaction and for between sample-sets normalization.

**Table 6:** Primer sequences and PCR conditions.

mRNA		primer sequence	Denaturing temperature and time °C (s)	Annealing temperature and time °C (s)	Elongation temperature and time °C (s)	Number of cycles	Product size (bp)
*NDFUV1*	S	5′-TACATCCGAGGGGAATTCTACA-3′	94 (60)	60 (60)	72 (60)	35	426
	NS	5′-GTTCTTTCAAGGGCACAGACAT-3′					
*NDUFV2*	S	5′-GGAGGAGCTTTATTTGTGCAC-3′	94 (60)	55 (60)	72 (60)	35	640
	NS	5′-CCTGCTTGTACACCAAATCC-3′					
*NDUFS1*	S	5′- TACTCGCTGCATCAGGTTTG-3′	94 (60)	58 (60)	72 (60)	35	299
	NS	5′-CATGCATACGTGGCAAAATC-3′					
β-actin	S	5′-TGAAGTGTGACGTGGACATCCG-3′	94 (60)	60 (60)	72 (60)	25	447
	NS	5′-GCTGTCACCTTCACCGTTCCAG-3′					
18S-RNA	S	5′-AGGAATTGACGGAAGGGCAC-3′	94 (60)	60 (60)	72 (60)	25	324
	NS	5′-GTGCAGCCC CGGACATCTAAG-3′					

### Immunoblotting

Protein isolated from frozen specimens was analyzed by immunoblotting three times for each individual [26]. Protein samples were separated on SDS-PAGE. Primary customized recombinant rabbit antihuman antibodies used; anti-51-kDa 1∶500, anti-24-kDa 1∶1500, anti-75-kDa 1∶1000 synthesized by Sigma-Aldrich, Israel. Secondary antibodies used; anti rabbit-IgG 1∶15,000 for 24-kDa, and 1∶10,000 for 51-k-Da and 75-kDa subunits (Santa Cruz Biotechnology Santa Cruz, CA). β-actin was used for normalizing variations in protein aliquots, as its levels were not affected by disease. In addition, a single batch of rat brain mitochondrial protein in three different concentrations was used as a positive control and for between sample-sets normalization.

### Statistical analysis

Normal distribution of data was analyzed by Kolmogorov-Smirnov test. For further analysis parametric tests were used, as most data (90%) presented normal distribution. For data not distributed normally, non-parametric and parametric tests showed similar differences between groups. Data were analyzed by two-way ANOVA followed by Bonferroni post-hoc test. Age, gender, laterality, PMI, brain pH, disease duration, drug and alcohol abuse and medication were added as covariates and persistence of the significant difference in main effect between diagnostic groups was assessed by ANCOVA. Correlations were analyzed by Pearson correlation test. SPSS version 14.0 software was used.

## Results

**Figure 1:**
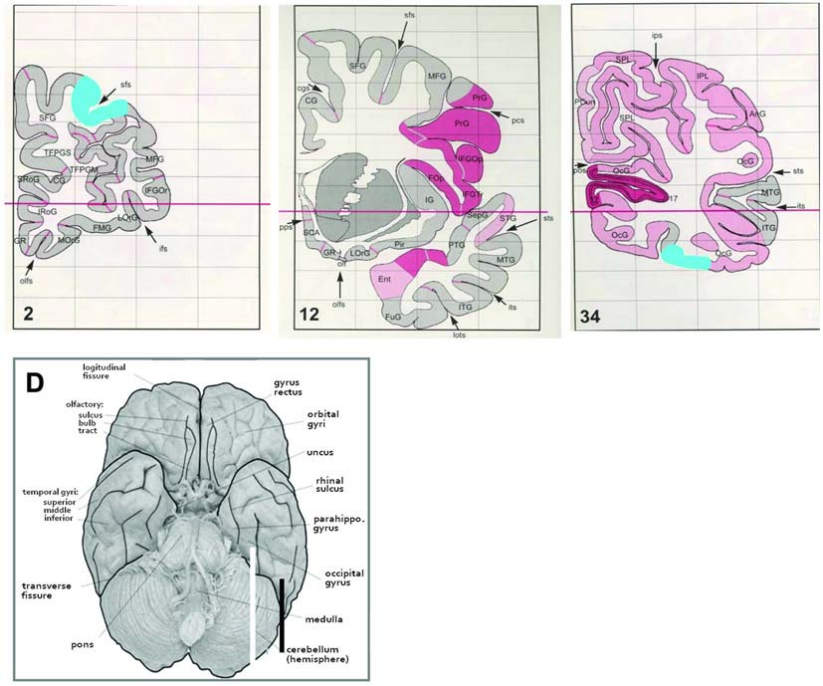
Diagrams of brain section presenting the four different brain areas in which complex I subunits were assessed. A) The prefrontal cortex (BA 9/46) sections are 14 µm frozen coronal sections though the area marked in blue B) The striatum sections are 14 µm frozen coronal sections through the head of the caudate nucleus and putamen at the level of the nucleus accumbens. C) The parieto-occipital cortex (BA 19) sections are 14 µm frozen coronal sections though the area marked in blue. D) The cerebellar sections are 14 um frozen sagittal sections through the lateral cerebellar hemisphere at the level marked by the green line. Diagram are obtained from the Atlas of the Human Brain by Jurgen K. Mai, Joseph Assheuer and George Paxinos, 1997 3^rd^ Ed. pp. 123, 124, 126 Elsevier Ltd.

Protein and mRNA levels of complex I subunits, 51-, 24- and 75-kDa, were analyzed in two brain specimens obtained from the striatum and the lateral cerebellar hemisphere (Fig 1) of patients with schizophrenia, bipolar disorder, major depression and normal subjects. In a previous study we have shown alterations in the same three subunits, in specimens obtained from the prefrontal (BA9/46) and the parieto-occipital (BA19) cortices of the same subject cohorts [26]. Results of the two studies were combined and analyzed by two-way ANOVA with the four subject cohorts and 4 brain areas as the two independent variables and mRNA and protein levels of 51-, 24- and 75-kDa as the dependent variables. The 75-kDa subunit was assessed in three brain regions, as parieto-occiptal cortex specimen was insufficient. A highly significant interaction between disease cohorts and brain areas for all dependent variables was observed (Table 7), suggesting that the different disease cohorts differentially affect complex I subunits expression in various brain areas.

**Table 7:** Two way ANOVA results of disease and brain area dependent alterations in complex I subunits.

	Dependent Variable	df	F	Sig.
Cohort	*NDUFV1*	3	8.131	0.000
	*NDUFV2*	3	11.297	0.000
	*NDUFS1*	3	9.646	0.000
	51-kDa	3	3.513	0.020
	24-kDa	3	5.663	0.001
	75-kDa	3	6.935	0.000
Area	*NDUFV1*	3	43.306	0.000
	*NDUFV2*	3	152.980	0.000
	*NDUFS1*	2	16.815	0.000
	51-kDa	3	368.109	0.000
	24-kDa	3	432.844	0.000
	75-kDa	2	399.590	0.000
Cohort*Area	*NDUFV1*	9	2.795	0.004
	*NDUFV2*	9	9.008	0.000
	*NDUFS1*	6	4.773	0.000
	51-kDa	9	7.148	0.000
	24-kDa	9	7.819	0.000
	75-kDa	6	4.295	0.000

The NDUFV1, NDUFV2 and NDUFS1 and 24-, 51- and 75-kDa stand for mRNA and protein levels of the three subunits of complex I, respectively. All subunits were analyzed in 4 brain areas, the striatum, the lateral hemisphere of the cerebellum, the prefrontal cortex (BA9/46) and the parieto-occipital cortex (BA19) in schizophrenia, bipolar disorder and major depression and normal subjects. The 75-kDa subunit was analyzed in all brain areas except the parieto-occipital cortex (BA19).

Alterations in complex I subunits in the prefrontal and the parieto-occipital cortices which were previously discussed [26] and are presented in Table 8. Data of complex I subunits' expression in the striatum and the cerebellum are further analyzed for disease related effects.

**Table 8:** Summary of mRNA and protein levels in four different brain areas.

	Striatum		Cerebellum		Prefrontal cortex (BA 46/9)		Parieto-occipital cortex (BA 19)	
**NDUFV1 (51 kDa)**								
	*mRNA*	*protein*	*mRNA*	*protein*	*mRNA*	*protein*	*mRNA*	*protein*
**Normal**	1.83±0.51	1.78±0.37	1.25±0.27	1.63±0.32	1.43±0.68	0.80±0.21	0.37±0.14	1.66±0.30
**SCH**	1.09±0.26 *	1.35±0.24 *	1.28±0.3	1.25±0.60	0.83±0.16 *	0.66±0.31 *	0.59±0.44*	1.92±0.17 *
**MD**	1.52±0.46	1.95±0.34	1.00±0.31 *	0.83±0.24 *	1.02±0.50	0.77±0.40	0.28±0.35*	1.73±0.28
**BP**	1.81±0.84	1.53±0.68	0.88±0.24 *	0.92±0.22 *	1.49±1.20	0.76±0.32	0.40±0.23	2.24±0.63 *
**NDUFV2 (24-kDa)**								
	*mRNA*	*protein*	*mRNA*	*protein*	*mRNA*	*protein*	*mRNA*	*protein*
**Normal**	1.24±0.32	1.03±0.22	1.59±0.48	1.04±0.17	0.50±0.07	0.89±0.24	0.31±0.17	1.35±0.18
**SCH**	0.67±0.18*	0.73±0.12 *	1.34±0.64	1.06±0.45	0.34±0.09*	0.39±0.19 *	0.66±0.37*	1.92±0.36 *
**MD**	1.23±0.81	0.93±0.19	0.85±0.30 *	0.57±0.22 *	0.43±0.16	0.49±0.31 *	0.29±0.28	1.5±0.32
**BP**	1.12±0.61	1.23±0.49	1.82±0.62	0.78±0.21	0.56±0.19	0.74±0.25	0.39±0.25	1.81±0.42 *
**NDUFS1 (75-kDa)**								
	*mRNA*	*protein*	*mRNA*	*protein*	*mRNA*	*protein*	*mRNA*	*protein*
**Normal**	1.27±0.58	2.63±0.52	0.82±0.19	1.40±0.13	1.17±0.69	1.08±0.19	ND	ND
**SCH**	0.55±0.16 *	2.61±0.33	0.73±0.50	1.04±0.34	1.14±0.32	1.01±0.13	ND	ND
**MD**	1.45±0.55	2.85±0.50	0.52±0.25*	0.95±0.29*	1.30±0.65	0.94±0.15	ND	ND
**BP**	1.09±0.56	2.20±0.27 *	0.62±0.14	0.97±0.23 *	0.92±0.54	0.98±0.19	ND	ND

Results are Mean±SD of arbitrary standardized densitometry values. *p<0.05 compared to control. MD-major depression, BP-bipolar, SCH- schizophrenic. (The data of the prefrontal and parieto-occipital cortices are calculated from the data published in Karry et al. 2002).

### Complex I subunits in the striatum

**Figure 2:**
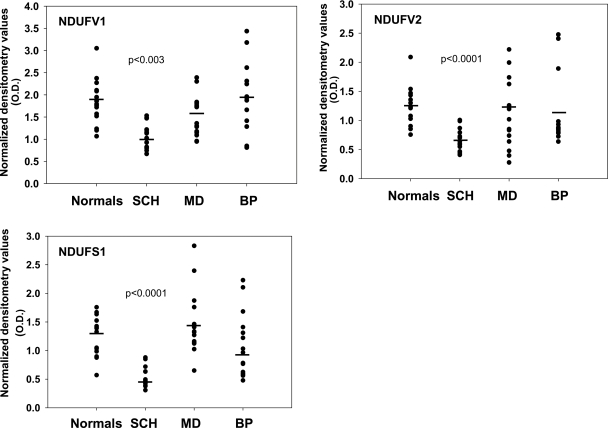
mRNA levels of NDUFV1, NDUFV2 and NDUFS1 subunits of complex I in post mortem striatum including the nucleus accumbens of patients with schizophrenia (SCH, n = 15), major depression (MD, n = 15) and bipolar disorder (BP, n = 15), and of normal controls (n = 15). Statistical significant differences vs. the control group were observed only in the schizophrenic group in all three subunits of complex I.

The significant difference in mRNA levels between the four subject cohorts for each subunit of complex I (NDUFV1-F(3,56) = 5.838, p = 0.002; NDUFV2-F(3,56) = 3.781, p = 0.015; NDUFS1-F(3,56) = 10.155, p = 0.0001) was due to a significant decrease in levels of NDUFV1 (41%; p = 0.003), NDUFV2 (46%; p = 0.0001),and NDUFS1 (60%; p = 0.0001) in the schizophrenic group as compared to controls. The bipolar and depressed groups did not differ from the controls. Interestingly, the schizophrenic group differed significantly from both mood disorder groups (NDUFV1, p = 0.004 vs. bipolar; NDUFV2, p = 0.03 vs. depressed; NDUFS1, p = 0.009 and p = 0.000 vs. bipolar and depressed, respectively). However, no difference was observed between depressed and bipolar patients (Fig 2).

**Figure 3:**
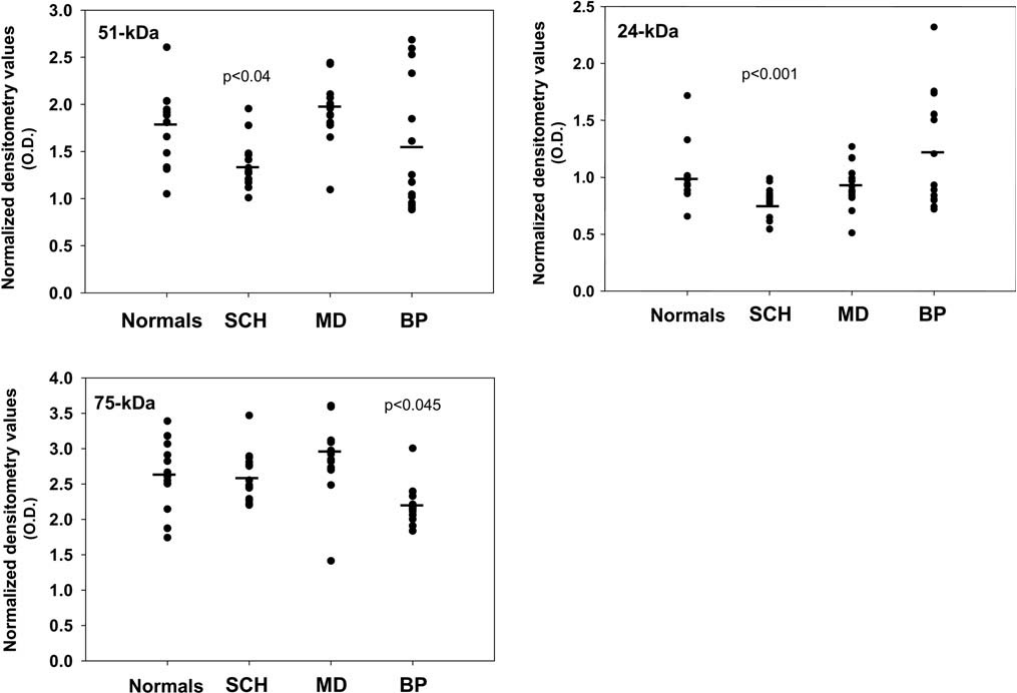
Protein levels of 51-, 24-and 75-kDa subunits of complex I in post mortem striatum including the nucleus accumbens of patients with schizophrenia (SCH, n = 15), major depression (MD, n = 15) and bipolar disorder (BP, n = 15) and of normal controls (n = 15). Statistical significant differences vs. the control group were observed in the 51- and 24-kDa subunits of the schizophrenic group and in the 75-kDa subunit of the bipolar group. No statistically significant difference was observed in the depressed group.

Protein levels of the 51-kDa (NDUFV1) and 24-kDa (NDUFV2) subunits, but not of 75-kDa (NDUFS1), showed a parallel pattern of change to that of the mRNA, throughout the four cohorts (F(3,56) = 5.604, p = 0.002, F(3,56) = 6.704, p = 0.001 and F(3,56) = 6.067, p = 0.001, respectively) (Fig 3). Thus, a significant decrease of 51-kDa (31%; p = 0.040) and 24-kDa (30%; p = 0.045) subunits, was observed in the schizophrenic group. While the depressed group did not differ from the controls for all three subunits, the bipolar group showed a slight, yet significant, decrease in the 75-kDa subunit (17%; p = 0.045). Similar to the findings in mRNA, the schizophrenic group differed significantly from both mood disorder groups (51-kDa, p = 0.002 vs. depressed; 24-kDa, p = 0.001 vs. bipolar; 75-kDa, p = 0.017 vs. bipolar). The bipolar and the depressed patients did not differ in 51-kDa and 24-kDa subunits but differed significantly in the 75-kDa subunit (p = 0.000).

### Complex I subunits in the cerebellum

**Figure 4:**
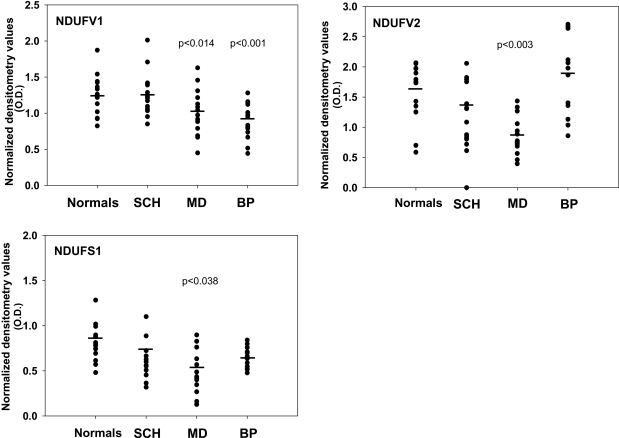
Cerebellar lateral hemisphere mRNA expression of complex I subunits. mRNA levels of NDUFV1, NDUFV2 and NDUFS1 subunits of complex I in post mortem cerebellar lateral hemisphere of patients with schizophrenia (SCH, n = 15), major depression (MD, n = 15) and bipolar disorder (BP, n = 15), and of normal controls (n = 15). Statistical significant differences vs. the control group were observed in all three subunits in the major depression group and in the NDUFV1 in the bipolar group. No statistically significant difference was observed in the schizophrenic group.

The lateral cerebellar hemisphere, which was planned to serve as a control area, showed interesting significant inter-group differences in mRNA of complex I subunits (NDUFV1-F(3,56) = 23.780, p = 0.0001, NDUFV2-F(3,56) = 12.234, p = 0.0001 and NDUFS1-F(3,56) = 4.331, p = 0.008). These changes were primarily due to the decrease in NDUFV1 (57%, p = 0.014), NDUFV2 (47%, p = 0.003) and NDUFS1 (36%, p = 0.038) in the depressed group as compared to controls, with less pronounced, but still significant, changes in the bipolar group in NDUFV1 (57%, p = 0.001) and NDUFS1 (30%), which did not reach significance (Fig 4). Unlike the findings in striatum, the schizophrenic group did not show significant changes from control in any of the three subunits. However, a significant difference was observed between the schizophrenic group and both groups with mood disorders. (NDUFV1, p = 0.000 vs. both bipolar and depressed; NDUFV2, p = 0.000 vs. depressed). No significant difference was observed between the depressed and the bipolar groups.

**Figure 5:**
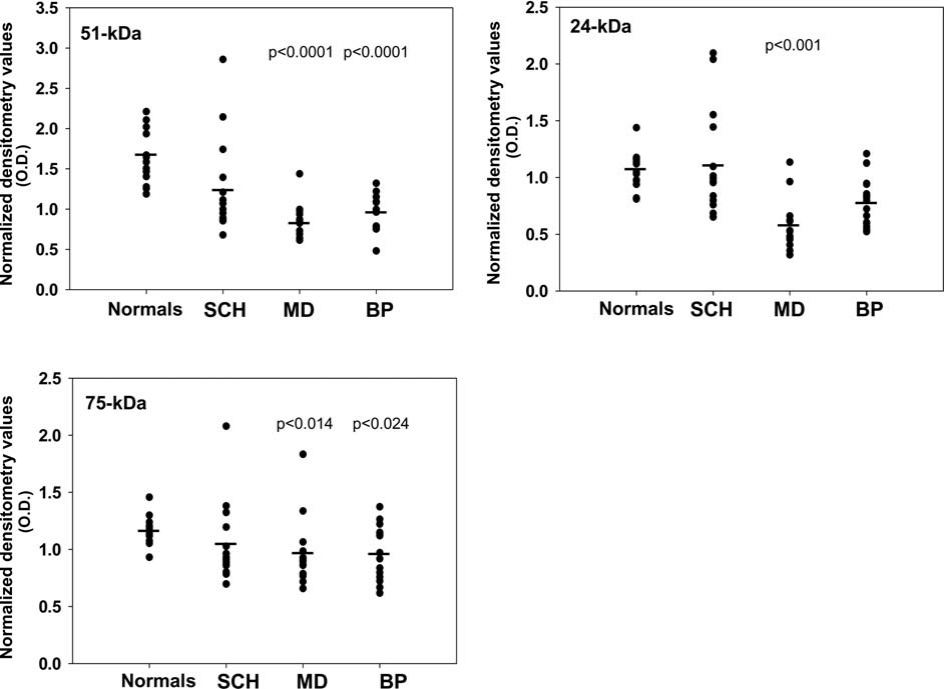
Cerebellar lateral hemisphere protein expression of complex I subunits. Protein levels of the 51-, 24-and 75-kDa subunits of complex I in post mortem Cerebellar lateral hemisphere of patients with schizophrenia (SCH, n = 15), major depression (MD, n = 15) and bipolar disorder (BP, n = 15), and of normal controls (n = 15). Statistical significant differences vs. the control group were observed in all three subunits in the major depression group and in the 51-kDa and the 75-kDa subunits in the bipolar group. No statistically significant difference was observed in the schizophrenic group.

Protein levels showed a parallel pattern of change to that of mRNA in the lateral cerebellar hemisphere (51-kDa-F(3,56) = 13.833, p = 0.0001; 24-kDa-F(3,56) = 8.044, p = 0.0001; 75-kDa-F(3,56) = 4.331, p = 0.008) (Fig 5). Thus, a significant decrease in the levels of the 51-kDa (43%, p = 0.001) and 75-kDa (30% p = 0.024) subunits was observed in the bipolar group and of 51-, 24- and 75-kDa subunits (49%, p = 0.0001, 45%, p = 0.001, 32% p = 0.014, respectively) in the depressed group. The schizophrenic group showed no significant difference from the control group in any of the subunits, similar to its mRNA findings, but was significantly different from the depressed group (51-kDa, p = 0.015; 24-kDa, p = 0.001). No significant difference was observed between the bipolar group and both the depressed or the schizophrenic group.

### Demographic parameters, covariance analysis

To control for potential confounds, age, gender, PMI, brain pH, side of brain, duration of disease, age of onset, severity of alcohol and drug abuse and psychotropic medication were added as covariates, and assessed by ANCOVA for all three subunits in the striatum and in the cerebellum. Severity of alcohol and drug abuse was scored from 0-no use to 6-heavy use. Disease related significant differences, observed in mRNA and protein levels of 51-, 24- or 75-kDa subunits, were not altered by any of the parameters used as covariant in all groups, including pH, alcohol and drug abuse and lifetime antipsychotic dose, in both brain areas for all patient groups, except for protein and mRNA levels of the 75-kDa, in which onset and duration of illness obliterated the disease related significance, but showed no significant correlation with 75-kDa mRNA or protein levels in any patient group. Table 8 presents disease-related adjusted means±SD of mRNA and protein after ANCOVA, for all three genes in the four cohorts.

### Effect of antipsychotic medication

Given the reported effects of antipsychotic drugs on brain energy metabolism in motor areas, specifically in the basal ganglia [42]–[44], together with their inhibitory effect on complex I activity [30], [33], [45], we redefined our group variable to define 2 subgroups; schizophrenic or bipolar patients medicated (12 schizophrenic, 7 bipolar patients) and unmedicated ( 3 schizophrenic, 8 bipolar patients) with antipsychotic drugs. Three schizophrenic patients and 3 bipolar patients were medication free and the other 5 bipolar patients received other psychotrophic drugs. No significant difference was observed between the two subgroups in mRNA or protein levels of all three subunits in the striatum and the cerebellum. In addition, there was no significant correlation between antipsychotic dose, expressed in fluphenazine milligram equivalents, and mRNA or protein of any of the three subunits in the antipsychotic medicated patients.

### Effect of antidepressant medication

To asses possible effects of antidepressant medications on complex I subunits in depressed subjects, we divided the depressed group into 2 subgroups: subjects medicated with antidepressant drugs (n = 9) and unmedicated (n = 6) subjects (two of them treated with other psychotropic drugs). No significant difference between groups was observed in mRNA or protein levels of all three subunits in the two brain areas.

### Effect of mood stabilizers

It was recently reported that lithium can affect genes of the mitochondrial OXPHOS [46]. To asses possible effects of mood-stabilizers on complex I subunits, we divided the bipolar group into 2 subgroups: subjects medicated with mood-stabilizers (n = 10) and umedicated (n = 5) subjects, 2 of them treated with other psychotropic drugs. No significant difference was observed between the two subgroups in mRNA or protein of all three subunits in both brain areas. We then redefined our group variable to include 2 subgroups: subjects receiving mood-stabilizers (n = 16; 10 bipolar, 3 depressed, 3 schizophrenics) and subjects not receiving mood-stabilizers (n = 44), again there was no significant difference between groups in both mRNA and protein of all three subunits in the two brain areas.

## Discussion

This study compares mRNA and protein levels of three subunits of mitochondrial complex I in different brain areas of schizophrenic, bipolar and depressed patients and normal subjects. The main finding of the present study is that complex I subunits are altered in all three psychiatric disorders, albeit in a disease specific neuroanatomical pattern. In schizophrenia, but not in affective disorders, a selective reduction in the 51- and 24-kDa subunit expression was observed in the striatum. However, in both affective disorders, reductions in complex I subunits were observed specifically in the cerebellum, with the depressed group demonstrating more consistent alterations. Thus, the depressed patients showed significant reductions in mRNA and protein of all three subunits of complex I, while in the bipolar group mRNA and protein of the 24-kDa subunit and only protein levels of the 75-kDa subunit were reduced, with no significant changes in the 51-kDa subunit. The phenomenon of a disease-specific regional distribution of aberrant expression of complex I subunits was also observed in our previous study on the same subject cohorts [26], demonstrating a schizophrenia-specific reduction in the 51- and 24-kDa expression in the prefrontal cortex (BA 46/9). In the parieto-occipital cortex (BA19), however, an increased expression of these subunits was observed both in schizophrenic and bipolar patients. In the depressed group, no consistent change was observed in both brain areas. Interestingly, similar to schizophrenia, the bipolar group demonstrated some abnormality in the striatum, as the 75-kDa protein levels were decreased in this area. Taken together, the results of both studies indicate that while the schizophrenic and depressed groups display disparate regional distribution of complex I alterations, the bipolar group shares considerable similarities with both the schizophrenic and depressed groups. The latter is in line with the significant overlap among patients with bipolar disorder and patients with schizophrenia or major depression in clinical symptoms, neurocognitive dysfunction, cerebral metabolism abnormalities, as well as brain biochemical and molecular pathophysiological processes [47]–[50].

Psychotropic medication can conceivably contribute to disease-specific regional alterations in complex I, as well as to the anatomical overlap the bipolar group displays with both the schizophrenic and depressed group. Indeed, it is well established that antipsychotics, typical and atypical, interact with the mitochondrial OXPHOS, specifically with complex I, directly inhibiting its activity, both *in vitro* and *in vivo* in rodents and humans [29], [30], [33], [51]. In addition, mitochondria are a target for mood stabilizers such as lithium and valproate [52] and for antidepressant drugs [53], primarily MAO inhibitors. In the present study, however, we were unable to detect any effect of antipsychotic, antidepressant or mood stabilizing medication on the disorder-related differences in mRNA or protein levels of complex I subunits. Moreover, no differential effect on complex I subunits could be observed upon dividing patients according to the type of medication they were receiving at time of death. Although these results should be taken with caution due to small sample size, this lack of effect of antipsychotic drugs is in line with our previous finding in platelets of schizophrenic patients [27], which suggests that while complex I activity is affected by antipsychotic medication, its subunits' mRNA and protein expression is not, a finding recently corroborated by our recent findings in the neonatal ventral hippocampus lesion rat model of schizophrenia [54] and unpublished data in neuronal cell line.

Another extensively addressed confounding factor in postmortem brain studies is sample pH [36], [55], [56]. Although the mechanism by which pH affects the expression of genes is unclear, it has been reported that the expression pattern of some genes differs between low and high pH-brain specimens [36], [55]. However, adding pH as a covariate did not change disease-related effects on complex I, which is in line with our previous study [26] and with its lack of effect on other genes such as glucocorticoid receptor, cytosolic protein kinase Cε and kainate receptor 2 [57]. Other covariates such as age, gender, alcohol and drug abuse, laterality and PMI had no effect on the statistical significance of the differences between the diagnostic groups. These results, with the reservation that small sample size may contribute to lack of confounders' effect, minimize the potential effect of possible artifacts due to postmortem analysis, giving further credence to disease-dependent main effects.

The present study examined three nuclear DNA encoded subunits of complex I, the 51- and 24-kDa subunits, two iron-sulfur flavoproteins with catalytic properties including the site for transhydrogenation from NADH to NAD^+^, and the 75-kDa, the largest iron-sulfur transmembranous subunit [58]. All three subunits form one functional subunit with a stoichiometry of 1mol of each subunit for 1mol of complex [59]. Therefore, it is plausible that any deviation from this ratio can lead to abnormal complex I activity. Indeed, we have previously shown that abnormal expression of both the 51- and 24-kDa with no change in the 75-kDa subunit, were associated with impaired activity of complex I in platelets of schizophrenic patients [27]. Since complex I activity measurement in whole tissue has low sensitivity and postmortem delay significantly affects it [60], direct assessment of brain complex I activity is relatively scarce. Taken together, the data suggest that complex I subunits' expression may reflect abnormalities in complex I activity.

In schizophrenic patients we observed a decrease in complex I subunits in prefrontal cortex and striatum, key elements in the cortico–striatal-thalamic circuitry modulating cognitive processes prominent in schizophrenia [61], and critically important in biological processes believed to underlie psychotic symptoms [62], [63]. Congruently, most imaging studies report abnormal metabolic activity of the cortico–striatal-thalamic circuitry in schizophrenia [63]. In the parieto-occipital cortex, however, increased levels of complex I subunits have been observed in both schizophrenic and bipolar patients, in line with previous findings of increased brain metabolic rates in both disorders [64]. The parieto-occipital cortex was shown to be involved in cognitive processes of mental visual imagery [65]. Aberrations in the ability to distinguish between perception and mental imagery has been suggested to be associated with psychotic hallucinations and paranoid delusions, characteristic of both disorders [66]–[68]. Interestingly, in the depressed group, alterations in both prefrontal and parieto-occipital cortices were either sporadic or not expressed at the functional relevant (protein) level.

The results of this study suggest the lateral hemisphere of the cerebellum as a prominent anatomical substrate for depression, as most consistent alterations were observed in the depressed group but also in the bipolar patients, as expected by the overlap of symptoms. The role of the cerebellum has traditionally been limited to coordination of voluntary movement, gait, posture, speech and motor function. However, evidence from studies of patients with overt cerebellar diseases as well as from normal subjects, suggests a possible role for the cerebellum in cognition, mood and behavior [69], [70]. For example, cerebellar increase of blood flow is often seen in normal subjects performing cognitive tasks and exposed to sadness evoking challenges. Several studies have shown that patients with various cerebellar pathologies demonstrate flattening of affect or have higher depression scores than control subjects [70], [71]. Furthermore, in patients with depression, transient mood challenges produced less activation in the cerebellum, prefrontal and limbic areas, than in healthy subjects [71]–[73]. Interestingly, it was reported that refractory depression responded to treatment with a chronically implanted cerebellar pacemaker [74]. In line with the suggested role of the cerebellum in mood and behavior is the abundance of serotonergic and noradrenergic inputs to the cerebellum, and their ability to modulate the cerebellar circuitry and affect cerebellar learning and control mechanisms [75].

The four brain areas we have analyzed are part of the neuronal circuitries implicated in all three mental disorders. Structural and functional imaging studies have implicated the cerebellum as part of the cortico-thalamic-cerebellar-cortical circuit, in schizophrenia [70], [71]. In this study, however, complex I subunits expression was unaltered in the lateral cerebellar hemisphere of the schizophrenic group. Similarly, the prefrontal cortex has been implicated in depression and in bipolar disorder, but alterations in complex I were either sporadic or absent. This apparent contradiction may be attributed to methodological differences, as imaging studies usually sample a broader brain area and do not exclude circuit-dependent inductions during measurement, whereas molecular studies are more localized.

Finally, in the present and previous studies we have shown a particular molecular pathology, in four discrete brain areas, and detected disease-specific pattern of regional alterations. Given the major role complex I plays in controlling mitochondrial OXPHOS its abnormal activity can result in mitochondrial dysfunction. Mitochondria, being the main source of high energy intermediates, are of prominent importance in maintaining the cellular energy state, particularly of high-energy consuming cells such as neurons. Hence, we hypothesize that mitochondrial dysfunction and thereby impaired neuronal metabolism can lead to alterations in neuronal function, plasticity and brain circuitry. Mitochondrial dysfunction can be either a causal or a consequential event of abnormal signaling in specific brain circuitries. Previous studies utilizing pharmacological tools to inhibit mitochondria, revealed defects in synaptic potentiation and a failure to maintain neurotransmission under rigorous stimulation [76], [77]. Further evidence for the critical role of mitochondria in neuronal activity is the finding that loss of mitochondria from axon terminals result in defective synaptic transmission in *Drosophila* [78]–[80] and that dendritic mitochondria are essential in the morphogenesis and plasticity of spines and synapses in hippocampal tissue slices [81], [82]. Our findings of mitochondrial impairment in peripheral blood cells in schizophrenia may support the suggestion of mitochondrial dysfunction as a primary event [24], [27], [83]. However, an opposite interaction between neuronal transmission and mitochondria was also demonstrated by the ability of neurotransmitters, mainly glutamate but also dopamine, to reciprocally affect mitochondrial function and ATP production process in a cellular system as well as in-vivo [84]–[89]. Given this reciprocal interaction between both, the question of primacy still remains an enigma.

Prefrontal cortex, parieto-occipital cortex, striatum and cerebellum may constitute one, or part of several complex neuronal circuits critical for optimal functioning of normal cognition, emotion and behavior. Considering our data as well as previously reported data such as those on the differential anatomical apolipoprotein D (apoD) abnormalities in schizophrenia and bipolar disorder [90], it is tempting to suggest that the diversity in the clinical spectrum of mental disorders may, at least in part, be attributed to the different anatomical pattern of specific impairments in the neuronal circuits implicated in the disorders.

## References

[1] Fujimoto T, Nakano T, Takano T, Hokazono Y, Asakura T (1992). Study of chronic schizophrenics using 31P magnetic resonance chemical shift imaging.. Acta Psychiatr Scand.

[2] Volz HR, Riehemann S, Maurer I, Smesny S, Sommer M (2000). Reduced phosphodiesters and high-energy phosphates in the frontal lobe of schizophrenic patients: a 31P chemical shift spectroscopic-imaging study.. Biol Psychiatry.

[3] Jayakumar PN, Venkatasubramanian G, Keshavan MS, Srinivas JS, Gangadhar BN (2006). MRI volumetric and 31P MRS metabolic correlates of caudate nucleus in antipsychotic-naive schizophrenia.. Acta Psychiatr Scand.

[4] Jensen JE, Miller J, Williamson PC, Neufeld RW, Menon RS (2006). Grey and white matter differences in brain energy metabolism in first episode schizophrenia: 31P-MRS chemical shift imaging at 4 Tesla.. Psychiatry Res.

[5] Fukuzako H, Fukuzako T, Hashiguchi T, Kodama S, Takigawa M (1999). Changes in levels of phosphorus metabolites in temporal lobes of drug-naive schizophrenic patients.. Am J Psychiatry.

[6] Reddy R, Keshavan MS (2003). Phosphorus magnetic resonance spectroscopy: its utility in examining the membrane hypothesis of schizophrenia.. Prostaglandins Leukot Essent Fatty Acids.

[7] Madhavarao CN, Chinopoulos C, Chandrasekaran K, Namboodiri MA (2003). Characterization of the N-acetylaspartate biosynthetic enzyme from rat brain.. J Neurochem.

[8] Deicken RF, Johnson C, Pegues M (2000). Proton magnetic resonance spectroscopy of the human brain in schizophrenia.. Rev Neurosci.

[9] Kato T (2005). Mitochondrial dysfunction in bipolar disorder: from 31P-magnetic resonance spectroscopic findings to their molecular mechanisms.. Int Rev Neurobiol.

[10] Bertolino A, Frye M, Callicott JH, Mattay VS, Rakow R (2003). Neuronal pathology in the hippocampal area of patients with bipolar disorder: a study with proton magnetic resonance spectroscopic imaging.. Biol Psychiatry.

[11] Stork C, Renshaw PF (2005). Mitochondrial dysfunction in bipolar disorder: evidence from magnetic resonance spectroscopy research.. Mol Psychiatry.

[12] Coupland NJ, Ogilvie CJ, Hegadoren KM, Seres P, Hanstock CC (2005). Decreased prefrontal Myo-inositol in major depressive disorder.. Biol Psychiatry.

[13] Yildiz-Yesiloglu A, Ankerst DP (2006). Review of 1H magnetic resonance spectroscopy findings in major depressive disorder: a meta-analysis.. Psychiatry Res.

[14] Kato T, Takahashi S, Shioiri T, Inubushi T (1992). Brain phosphorous metabolism in depressive disorders detected by phosphorus-31 magnetic resonance spectroscopy.. J Affect Disord.

[15] Washizuka S, Kametani M, Sasaki T, Tochigi M, Umekage T (2006). Association of mitochondrial complex I subunit gene NDUFV2 at 18p11 with schizophrenia in the Japanese population.. Am J Med Genet B Neuropsychiatr Genet.

[16] Kato T, Kunugi H, Nanko S, Kato N (2001). Mitochondrial DNA polymorphisms in bipolar disorder.. J Affect Disord.

[17] Martorell L, Segues T, Folch G, Valero J, Joven J (2006). New variants in the mitochondrial genomes of schizophrenic patients.. Eur J Hum Genet.

[18] McMahon FJ, Chen YS, Patel S, Kokoszka J, Brown MD (2000). Mitochondrial DNA sequence diversity in bipolar affective disorder.. Am J Psychiatry.

[19] Middleton FA, Mirnics K, Pierri JN, Lewis DA, Levitt P (2002). Gene expression profiling reveals alterations of specific metabolic pathways in schizophrenia.. J Neurosci.

[20] Altar CA, Jurata LW, Charles V, Lemire A, Liu P (2005). Deficient hippocampal neuron expression of proteasome, ubiquitin, and mitochondrial genes in multiple schizophrenia cohorts.. Biol Psychiatry.

[21] Prabakaran S, Swatton JE, Ryan MM, Huffaker SJ, Huang JT (2004). Mitochondrial dysfunction in schizophrenia: evidence for compromised brain metabolism and oxidative stress.. Mol Psychiatry.

[22] Ben-Shachar D (2002). Mitochondrial dysfunction in schizophrenia: a possible linkage to dopamine.. J Neurochem.

[23] Ben-Shachar D, Laifenfeld D (2004). Mitochondria, synaptic plasticity, and schizophrenia.. Int Rev Neurobiol.

[24] Ben-Shachar D, Zuk R, Gazawi H, Reshef A, Sheinkman A (1999). Increased mitochondrial complex I activity in platelets of schizophrenic patients.. Inter J Neuropsychopharmacol.

[25] Maurer I, Zierz S, Moller H (2001). Evidence for a mitochondrial oxidative phosphorylation defect in brains from patients with schizophrenia.. Schizophr Res.

[26] Karry R, Klein E, Ben Shachar D (2004). Mitochondrial complex I subunits expression is altered in schizophrenia: a postmortem study.. Biol Psychiatry.

[27] Dror N, Klein E, Karry R, Sheinkman A, Kirsh Z (2002). State dependent alterations in mitochondrial complex I activity in platelets: A potential peripheral marker for schizophrenia.. Mol Psychiatry.

[28] Mulcrone J, Whatley SA, Ferrier IN, Marchbanks RM (1995). A study of altered gene expression in frontal cortex from schizophrenic patients using differential screening.. Schizophr Res.

[29] Whatley SA, Curi D, Das Gupta F (1998). Superoxide, neuroleptics and the ubiquinone and cytochrome b5 reductases in brain and lymphocytes from normals and schizophrenic patients.. Mol Psychiatry.

[30] Maurer I, Moller HJ (1997). Inhibition of complex I by neuroleptics in normal human brain cortex paralles the extrapyramidal toxicity of neuroleptics.. Mol Cell Biochem.

[31] Cavelier L, Jazin E, Eriksson I, Prince J, Bave B (1995). Decreased cytochrome c oxidase activity and lack of age related accumulation of mtDNA in brain of schizophrenics.. Genomics.

[32] Prince JA, Blennow K, Gottfries CG, Karlsson I, Oreland L (1999). Mitochondrial function in differentially altered in the basal ganglia of chronic schizophrenics.. Neuropsychopharmacol.

[33] Burkhardt C, Kelly JP, Lim YH, Filley CM, Parker WD (1993). Neuroleptic medications inhibit complex I of the electron transport chain.. Ann Neurol.

[34] Mehler-Wex C, Duvigneau JC, Hartl RT, Ben-Shachar D, Warnke A (2006). Increased mRNA levels of the mitochondrial complex I 75-kDa subunit : A potential peripheral marker of early onset schizophrenia?. Eur Child Adolesc Psychiatry.

[35] Konradi C, Eaton M, MacDonald ML, Walsh J, Benes FM (2004). Molecular evidence for mitochondrial dysfunction in bipolar disorder.. Arch Gen Psychiatry.

[36] Iwamoto K, Bundo M, Kato T (2005). Altered expression of mitochondria-related genes in postmortem brains of patients with bipolar disorder or schizophrenia, as revealed by large-scale DNA microarray analysis.. Hum Mol Genet.

[37] Vawter MP, Tomita H, Meng F, Bolstad B, Li J (2006). Mitochondrial-related gene expression changes are sensitive to agonal-pH state: implications for brain disorders.. Mol Psychiatry.

[38] Gardner A, Johansson A, Wibom R, Nennesmo I, von Dbeln U (2003). Alterations of mitochondrial function and correlations with personality traits in selected major depressive disorder patients.. Journal of Affective Disorders.

[39] Davey GP, Peuchen S, Clark JB (1998). Energy thresholds in brain mitochondria: potential involvement in neurodegeneration.. J Biol Chem.

[40] Torrey EF, Webster M, Knable M, Johnston N, Yolken RH (2000). The stanley foundation brain collection and neuropathology consortium.. Schizophr Res.

[41] Laifenfeld D, Karry R, Klein E, Ben-Shachar D (2005). Alterations in cell adhesion molecule L1 and functionally related genes in major depression: a postmortem study.. Biol Psychiatry.

[42] Buchsbaum MS, Potkin SG, Siegel BV, Lohr J, Katz M (1992). Striatal metabolic rate and clinical response to neuroleptics in schizophrenia.. Arch Gen Psychiatry.

[43] Desco M, Gispert JD, Reig S, Sanz J, Pascau J (2003). Cerebral metabolic patterns in chronic and recent-onset schizophrenia.. Psychiatry Res.

[44] Corson PW, O'Leary DS, Miller DD, Andreasen NC (2002). The effects of neuroleptic medications on basal ganglia blood flow in schizophreniform disorders: a comparison between the neuroleptic-naive and medicated states.. Biol Psychiatry.

[45] Barrientos A, Marin C, Miro O, Casademont J, Gomez M (1998). Biochemical and molecular effects of chronic haloperidol administration on brain and muscle mitochondria of rats.. J Neurosci Res.

[46] Sun X, Wang JF, Tseng M, Young LT (2006). Downregulation in components of the mitochondrial electron transport chain in the postmortem frontal cortex of subjects with bipolar disorder.. J Psychiatry Neurosci.

[47] Carlson PJ, Singh JB, Zarate J, Carlos A, Drevets WC, Manji HK (2006). Neural Circuitry and Neuroplasticity in Mood Disorders: Insights for Novel Therapeutic Targets.. NeuroRX New Directions in Psychiatric Therapeutics.

[48] Daneluzzo E, Arduini L, Rinaldi O, Di Domenico M, Petruzzi C (2002). PANSS factors and scores in schizophrenic and bipolar disorders during an index acute episode: a further analysis of the cognitive component.. Schizophr Res.

[49] Dunn RT, Kimbrell TA, Ketter TA, Frye MA, Willis MW (2002). Principal components of the Beck Depression Inventory and regional cerebral metabolism in unipolar and bipolar depression.. Biol Psychiatry.

[50] Manji HK, Lenox RH (2000). Signaling:cellular insights into the pathpysiology of bipolar disorder.. Biol Psychiatry.

[51] Prince JA, Yassin MS, Oreland L (1997). Neuroleptic-induced mitochondrial enzyme alterations in the rat brain.. J Pharmacol Exper Ther.

[52] Kato T (2007). Mitochondrial dysfunction as the molecular basis of bipolar disorder : therapeutic implications.. CNS Drugs.

[53] Weinbach EC, Costa JL, Nelson BD, Claggett CE, Hundal T (1986). Effects of tricyclic antidepressant drugs on energy-linked reactions in mitochondria.. Biochem Pharmacol.

[54] Ben-Shachar D, Nadri C, Karry R, Agam G (2008). Mitochondrial Complex I Subunits are Altered in Rats with Neonatal Ventral Hippocampal Damage but not in Rats Exposed to Oxygen Restriction at Neonatal Age.. J Mol Neurosci.

[55] Li JZ, Vawter MP, Walsh DM, Tomita H, Evans SJ (2004). Systematic changes in gene expression in postmortem human brains associated with tissue pH and terminal medical conditions.. Hum Mol Genet.

[56] Bahn S, Augood SJ, Ryan M, Standaert DG, Starkey M (2001). Gene expression profiling in the post-mortem human brain–no cause for dismay.. J Chem Neuroanat.

[57] Knable MB, Barci BM, Bartko JJ, Webster MJ, Torrey EF (2002). Molecular abnormalities in the major psychiatric illnesses: Classification and Regression Tree (CRT) analysis of post-mortem prefrontal markers.. Mol Psychiatry.

[58] Hatefi Y (1985). The mitochondrial electron transport and oxidative phosphorylation system.. Annu Rev Biochem.

[59] Belogrudov G, Hatefi Y (1994). Catalytic sector of complex I (NADH:ubiquinone oxidoreductase): subunit stoichiometry and substrate-induced conformation changes.. Biochemistry.

[60] Mizuno Y, Suzuki K, Ohta S (1990). Postmortem changes in mitochondrial respiratory enzymes in brain and a preliminary observation in Parkinson's disease.. J Neurol Sci.

[61] Harrison PJ, Weinberger DR (2005). Schizophrenia genes, gene expression, and neuropathology: on the matter of their convergence.. Mol Psychiatry.

[62] Chouinard G, Miller R (1999). A rating scale for psychotic symptoms (RSPS) part I: theoretical principles and subscale 1: perception symptoms (illusions and hallucinations).. Schizophr Res.

[63] Buchsbaum MS, Hazlett EA (1998). Positron emission tomography studies of abnormal glucose metabolism in schizophrenia.. Schizophr Bull.

[64] al-Mousawi AH, Evans N, Ebmeier KP, Roeda D, Chaloner F (1996). Limbic dysfunction in schizophrenia and mania. A study using 18F-labelled fluorodeoxyglucose and positron emission tomography.. Br J Psychiatry.

[65] Barnes J, Howard RJ, Senior C, Brammer M, Bullmore ET (2000). Cortical activity during rotational and linear transformations.. Neuropsychologia.

[66] Frith C, Dolan RJ (1997). Brain mechanisms associated with top-down processes in perception.. Philos Trans R Soc Lond B Biol Sci.

[67] Aleman A, Bocker KBE, Hijman R, Kahn RS (2002). Hallucinations in schizophrenia: imbalance between imagery and perception.. Schizophr Res.

[68] Baethge C, Baldessarini RJ, Freudenthal K, Streeruwitz A, Bauer M (2005). Hallucinations in bipolar disorder: characteristics and comparison to unipolar depression and schizophrenia.. Bipolar Disord.

[69] Schmahmann JD (2004). Disorders of the cerebellum: ataxia, dysmetria of thought, and the cerebellar cognitive affective syndrome.. J Neuropsychiatry Clin Neurosci.

[70] Rapoport M, van Reekum R, Mayberg H (2000). The role of the cerebellum in cognition and behavior: a selective review.. J Neuropsychiatry Clin Neurosci.

[71] Konarski JZ, McIntyre RS, Grupp LA, Kennedy SH (2005). Is the cerebellum relevant in the circuitry of neuropsychiatric disorders?. J Psychiatry Neurosci.

[72] Beauregard M, Leroux JM, Bergman S, Arzoumanian Y, Beaudoin G (1998). The functional neuroanatomy of major depression: an fMRI study using an emotional activation paradigm.. Neuroreport.

[73] Smith KA, Ploghaus A, Cowen PJ, McCleery JM, Goodwin GM (2002). Cerebellar responses during anticipation of noxious stimuli in subjects recovered from depression. Functional magnetic resonance imaging study.. Br J Psychiatry.

[74] Heath RG, Llewellyn RC, Rouchell AM (1980). The cerebellar pacemaker for intractable behavioral disorders and epilepsy: follow-up report.. Biol Psychiatry.

[75] Schweighofer N, Doya K, Kuroda S (2004). Cerebellar aminergic neuromodulation: towards a functional understanding.. Brain Res Brain Res Rev.

[76] Alnaes E, Rahamimoff R (1975). On the role of mitochondria in transmitter release from motor nerve terminals..

[77] Tang Y-g, Zucker RS (1997). Mitochondrial Involvement in Post-Tetanic Potentiation of Synaptic Transmission.. Neuron.

[78] Guo X, Macleod GT, Wellington A, Hu F, Panchumarthi S (2005). The GTPase dMiro is required for axonal transport of mitochondria to Drosophila synapses.. Neuron.

[79] Stowers RS, Megeath LJ, Gorska-Andrzejak J, Meinertzhagen IA, Schwarz TL (2002). Axonal transport of mitochondria to synapses depends on milton, a novel Drosophila protein.. Neuron.

[80] Verstreken P, Ly CV, Venken KJ, Koh TW, Zhou Y (2005). Synaptic mitochondria are critical for mobilization of reserve pool vesicles at Drosophila neuromuscular junctions.. Neuron.

[81] Greenwood SM, Mizielinska SM, Frenguelli BG, Harvey J, Connolly CN (2007). Mitochondrial Dysfunction and Dendritic Beading during Neuronal Toxicity..

[82] Li Z, Okamoto K-I, Hayashi Y, Sheng M (2004). The Importance of Dendritic Mitochondria in the Morphogenesis and Plasticity of Spines and Synapses.. Cell.

[83] Ben-Shachar D, Karry R (2007). Sp1 expression is disrupted in schizophrenia; a possible mechanism for the abnormal expression of mitochondrial complex I genes, NDUFV1 and NDUFV2.. PLoS ONE.

[84] Brenner-Lavie H, Klein E, Zuk R, Gazawi H, Ljubuncic P (2008). Dopamine modulates mitochondrial function in viable SH-SY5Y cells possibly via its interaction with complex I: relevance to dopamine pathology in schizophrenia.. Biochim Biophys Acta.

[85] White RJ, Reynolds IJ (1996). Mitochondrial depolarization in glutamate-stimulated neurons: an early signal specific to excitotoxin exposure.. J Neurosci.

[86] Przedborski S, Jackson-Lewis V, Muthane U, Jiang H, Ferreria M (1993). Chronic levodopa administration alters cerebral mitochondrial respiratory chain activity.. Ann Neurol.

[87] Chan P, Di Monte DA, Luo JJ, DeLanney LE, Irwin I (1994). Rapid ATP loss caused by methamphetamine in the mouse striatum: relationship between energy impairment and dopaminergic neurotoxicity.. J Neurochem.

[88] Berman SB, Hastings TG (1999). Dopamine oxidation alters mitochondrial respiration and induces permeability transition in brain mitochondria: implications for Parkinson's disease.. J Neurochem.

[89] Garcia O, Almeida A, Massieu L, Bolanos JP (2005). Increased mitochondrial respiration maintains the mitochondrial membrane potential and promotes survival of cerebellar neurons in an endogenous model of glutamate receptor activation..

[90] Thomas EA, Dean B, Scarr E, Copolov D, Sutcliffe JG (2003). Differences in neuroanatomical sites of apoD elevation discriminate between schizophrenia and bipolar disorder.. Mol Psychiatry.

[91] Afifi AK (2003). The basal ganglia: a neural network with more than motor function.. Semin Pediatr Neurol.

[92] Graybiel AM (2005). The basal ganglia: learning new tricks and loving it.. Curr Opin Neurobiol.

[93] Hauber W (1998). Involvement of basal ganglia transmitter systems in movement initiation.. Prog Neurobiol.

[94] Packard MG, Knowlton BJ (2002). Learning and memory functions of the Basal Ganglia.. Annu Rev Neurosci.

[95] Crinion J, Turner R, Grogan A, Hanakawa T, Noppeney U (2006). Language control in the bilingual brain.. Science.

[96] Kimura M (1995). Role of basal ganglia in behavioral learning.. Neurosci Res.

[97] Kalivas PW, Volkow ND (2005). The neural basis of addiction: a pathology of motivation and choice.. Am J Psychiatry.

[98] Di Chiara G, Bassareo V (2007). Reward system and addiction: what dopamine does and doesn't do.. Curr Opin Pharmacol.

[99] Morton SM, Bastian AJ (2007). Mechanisms of cerebellar gait ataxia.. Cerebellum.

[100] Thach WT (2007). On the mechanism of cerebellar contributions to cognition.. Cerebellum.

[101] Apps R, Garwicz M (2005). Anatomical and physiological foundations of cerebellar information processing.. Nat Rev Neurosci.

[102] Nitschke MF, Arp T, Stavrou G, Erdmann C, Heide W (2005). The cerebellum in the cerebro-cerebellar network for the control of eye and hand movements–an fMRI study.. Prog Brain Res.

[103] Jung RE, Haier RJ (2007). The Parieto-Frontal Integration Theory (P-FIT) of intelligence: converging neuroimaging evidence.. Behav Brain Sci.

[104] Blumenfeld RS, Ranganath C (2007). Prefrontal cortex and long-term memory encoding: an integrative review of findings from neuropsychology and neuroimaging.. Neuroscientist.

[105] Fuster JM (2000). Executive frontal functions.. Exp Brain Res.

[106] Owen AM (2000). The role of the lateral frontal cortex in mnemonic processing: the contribution of functional neuroimaging.. Exp Brain Res.

[107] Orban GA (2008). Higher order visual processing in macaque extrastriate cortex.. Physiol Rev.

[108] Merriam EP, Colby CL (2005). Active vision in parietal and extrastriate cortex.. Neuroscientist.

[109] Sincich LC, Horton JC (2005). The circuitry of V1 and V2: integration of color, form, and motion.. Annu Rev Neurosci.

[110] Ohnishi T (1998). Iron-sulfur clusters/semiquinones in Complex I.. Biochimica et Biophysica Acta (BBA) - Bioenergetics.

[111] Han A-L, Yagi T, Hatefi Y (1989). Studies on the structure of NADH:ubiquinone oxidoreductase complex: Topography of the subunits of the iron-sulfur protein component.. Archives of Biochemistry and Biophysics.

[112] Laugel V, This-Bernd V, Cormier-Daire V, Speeg-Schatz C, de Saint-Martin A (2007). Early-onset ophthalmoplegia in Leigh-like syndrome due to NDUFV1 mutations.. Pediatr Neurol.

[113] Benit P, Chretien D, Kadhom N, de Lonlay-Debeney P, Cormier-Daire V (2001). Large-scale deletion and point mutations of the nuclear NDUFV1 and NDUFS1 genes in mitochondrial complex I deficiency.. Am J Hum Genet.

[114] Schuelke M, Smeitink J, Mariman E, Loeffen J, Plecko B (1999). Mutant NDUFV1 subunit of mitochondrial complex I causes leukodystrophy and myoclonic epilepsy.. Nat Genet.

[115] Washizuka S, Kametani M, Sasaki T, Tochigi M, Umekage T (2006). Association of mitochondrial complex I subunit gene NDUFV2 at 18p11 with schizophrenia in the Japanese population.. Am J Med Genet B Neuropsychiatr Genet.

[116] Xu C, Li PP, Kennedy JL, Green M, Hughes B (2008). Further support for association of the mitochondrial complex I subunit gene NDUFV2 with bipolar disorder.. Bipolar Disord.

[117] Benit P, Beugnot R, Chretien D, Giurgea I, De Lonlay-Debeney P (2003). Mutant NDUFV2 subunit of mitochondrial complex I causes early onset hypertrophic cardiomyopathy and encephalopathy.. Hum Mutat.

[118] Swerdlow RH, Weaver B, Grawey A, Wenger C, Freed E (2006). Complex I polymorphisms, bigenomic heterogeneity, and family history in Virginians with Parkinson's disease.. J Neurol Sci.

